# Supplementary material for: Solitary Bees Host More Bacteria and Fungi on Their Cuticle than Social Bees
Source: Microorganisms. 2023 Nov 16;11(11):2780. doi: 10.3390/microorganisms11112780 (PMC10673014; doi:10.3390/microorganisms11112780)

Figure S1 - Full legend of the cuticular community of bacterial and fungal genera (please see Figure 2).

A

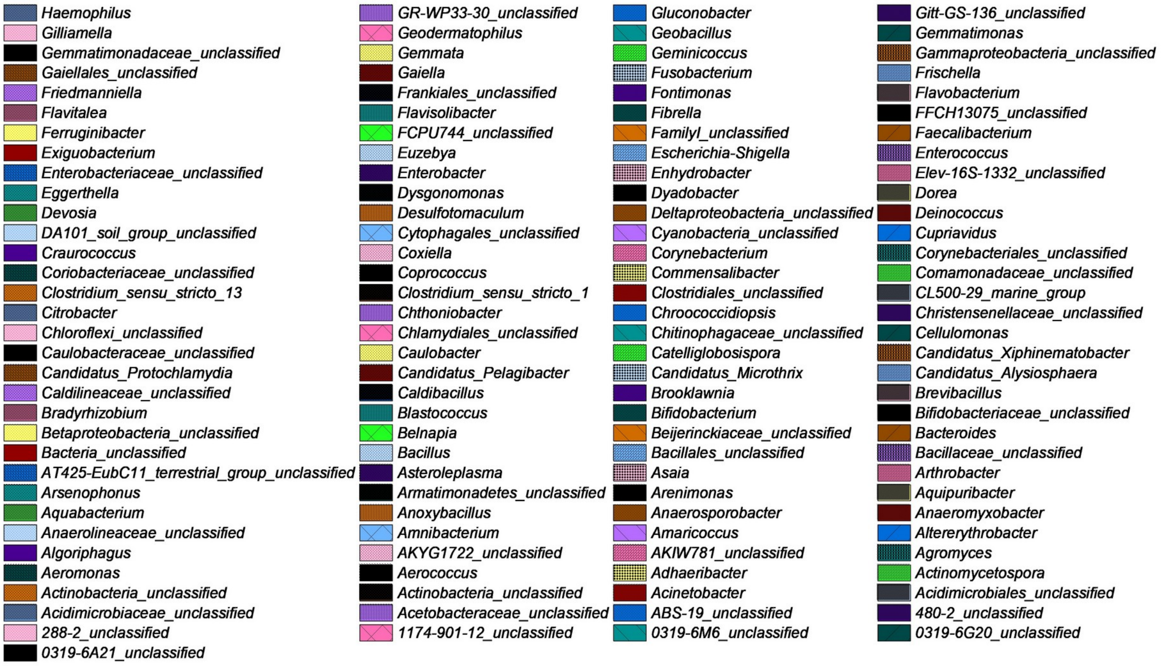

B

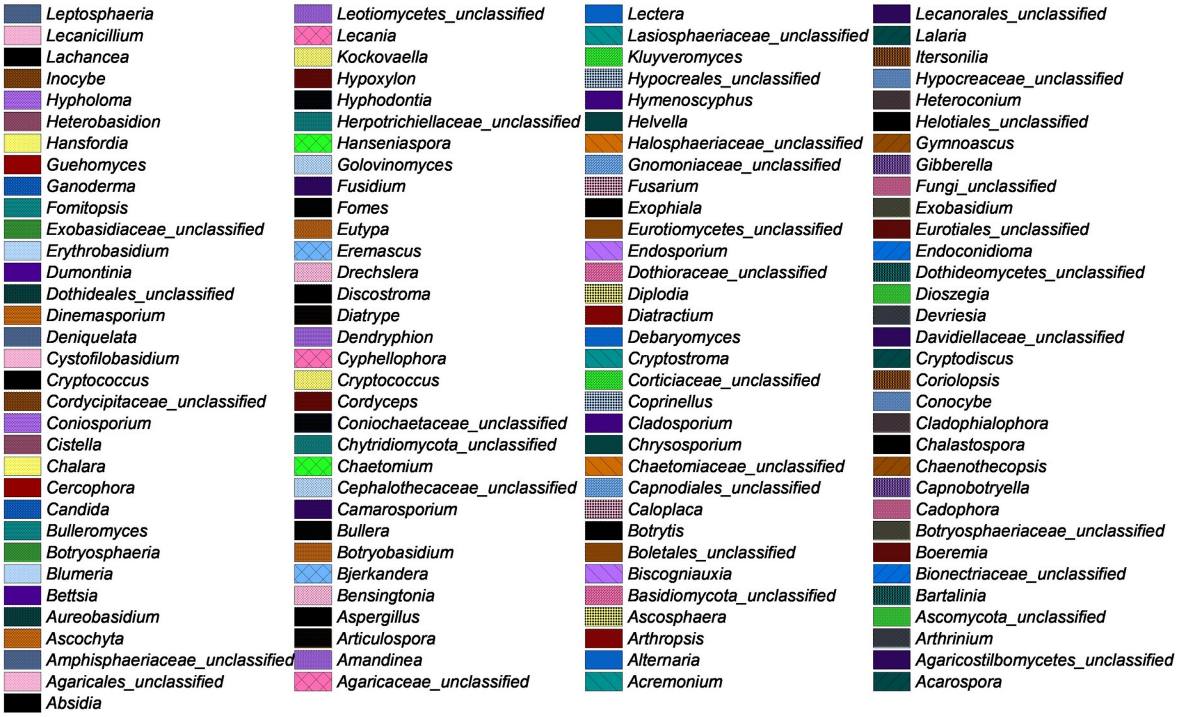

Figure S2 – Detrended correspondence analysis of the cuticle microbiome composition.

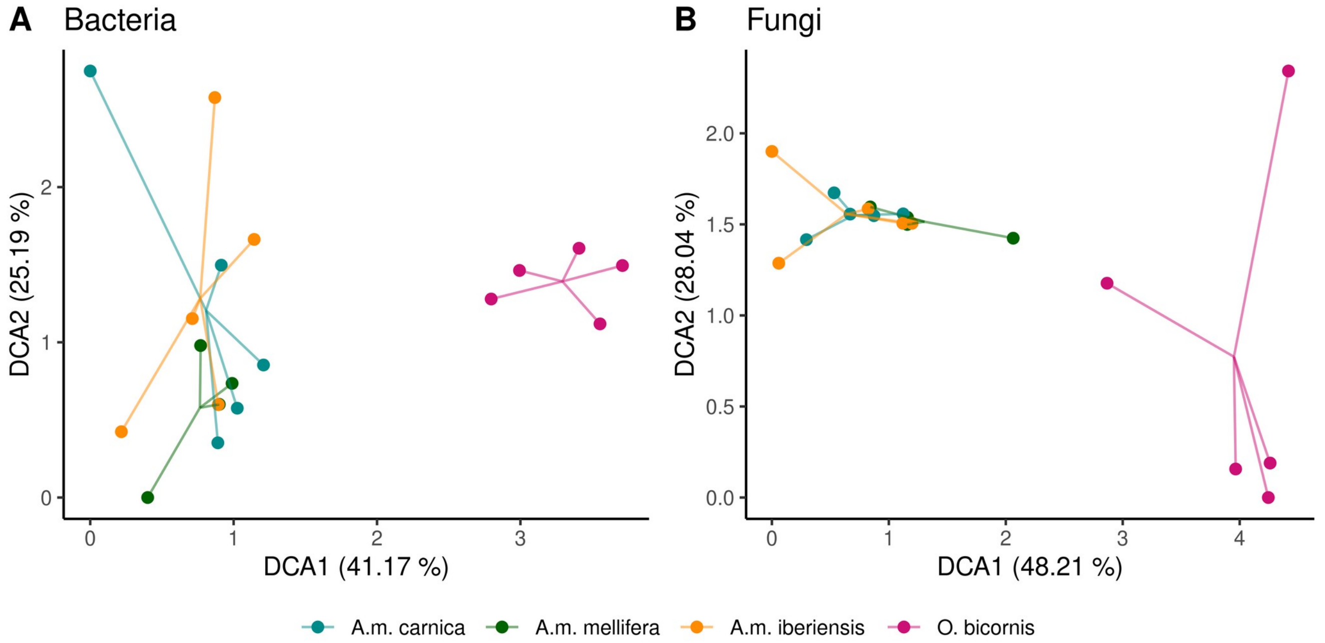

Figure S3 - Full legend of bacterial functions found in the cuticular bacterial communities (please see Figure 5).

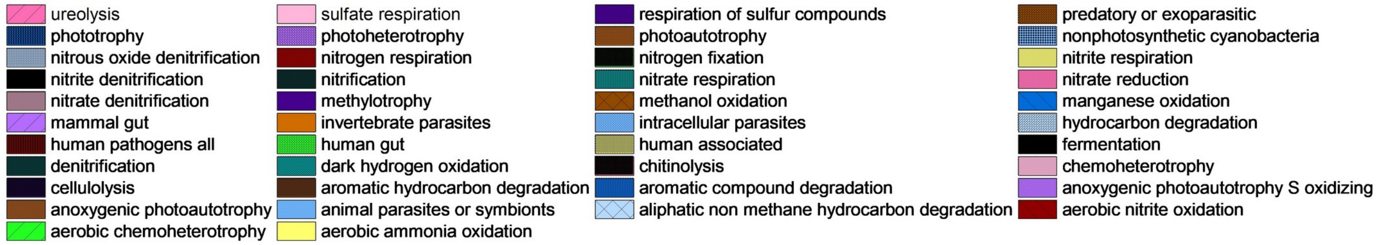

Supplement: Supplementary file 1 [file microorganisms-11-02780-s001.zip › microorganisms-2682838-supplementary.pdf]
